# Ataxia and Seizures despite Phenytoin: A Case Report Highlighting the Importance of TDM and Genetic Influences

**DOI:** 10.1155/2024/2888895

**Published:** 2024-03-20

**Authors:** Rachel Manoj, Arpita Meher, Jefry Winner G.

**Affiliations:** ^1^Faculty of Medicine, Nicolae Testemitanu State University of Medicine and Pharmacy, Chișinău, Moldova; ^2^Department of Medicine, Tbilisi State Medical University, Tbilisi, Georgia; ^3^JIPMER, Puducherry, India

## Abstract

Adverse drug reactions to commonly prescribed medications such as phenytoin, used for seizures, often go undetected due to various factors. This case report highlights a 52-year-old male diagnosed with late-onset epilepsy who was prescribed phenytoin. Despite the standard dosage, the patient experienced toxicity symptoms and a seizure, prompting admission for assessment. Laboratory tests and imaging were inconclusive, leading to a therapeutic drug monitoring (TDM) consultation, which revealed elevated phenytoin levels. Genetic testing for CYP2C9 polymorphisms was not feasible but noted as significant, especially in populations with higher prevalence. Phenytoin was tapered, leading to the patient's gradual recovery upon discontinuation and transition to valproate. The Naranjo scale predicted potential adverse drug responses (ADRs). This case underscores the significance of TDM, genetic considerations in drug metabolism, and the need to be vigilant in treating epilepsy to prevent such adverse events.

## 1. Introduction

Adverse drug reactions are more common than we think. Most of them are missed and underreported because of the high workload in hospitals. A study by Vallono et al. reported physicians perceived high workload, organizational activities, pharmacovigilance systems, and potential conflicts as major reasons for underreporting and missing ADRs [1]. In a systematic review by García-Abeijon et al., the leading cause for underreporting was nonobligation in reporting [2]. Phenytoin is one of the most commonly used and prescribed antiseizure drugs. Phenytoin has a narrow therapeutic margin and should be closely monitored. The primary concern and unpredictable scenario is when patients develop toxicity despite usual therapeutic doses (200–400 mg/day) [3]. The drug follows a nonlinear pharmacokinetic profile. Apart from that, the toxicity profile may also be attributable to pharmacogenetic changes in CYP2C9, which are essential for phenytoin metabolism. One of the well-documented evidence regarding the CYP2C9 polymorphisms is that it is highly prevalent in the Indian population [4]. In a study done in the Indian population by Chaudhary et al., CYP2C9^*∗*^3 (Leu359) polymorphism showed significantly higher serum phenytoin levels than the wild variant. The frequency was reported as high as 8% in this study [4]. Zhou et al. showed that CYP2C9^*∗*^2 is most prevalent in Europe and the Middle East, and CYP2C9^*∗*^3 is mostly responsible for reduced enzymatic activity and is abundant in South Asia [5]. Genetic polymorphism of CYP2C9 may reduce the metabolism of PHT by 25–50% in patients with variants ^*∗*^2 and ^*∗*^3 compared to those with wild-type variant ^*∗*^1 [6]. This signifies the importance of genetic testing, especially in South Asian and Indian patients whom we recommend to undergo genetic testing. However, TDM is underutilized, and genetic testing is expensive to afford in many healthcare settings. In this case report, we describe the presentation of a patient with phenytoin toxicity and emphasize the significance of TDM and genetic testing in feasible settings.

## 2. Case Presentation

**Figure 1:**
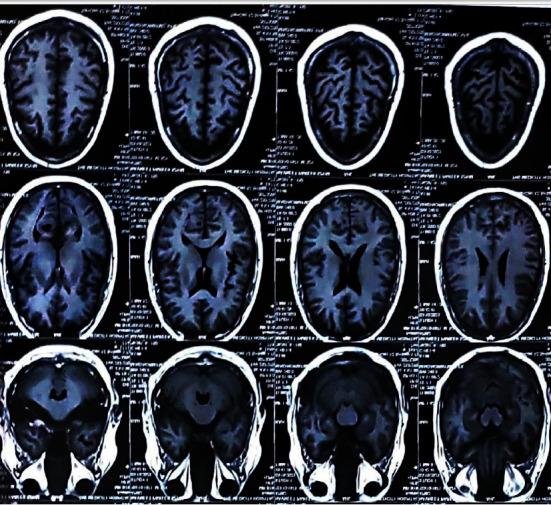
Normal study: MRI brain.

A 52-year-old male was diagnosed with “late onset epilepsy” and was prescribed 200 mg phenytoin twice daily. He had a focal seizure with impaired awareness and was on regular medication. The patient was not advised to undergo TDM. After one month of treatment, he could not walk and had frequent falls. He also had an attack of seizure despite being on phenytoin. The patient was well-oriented to time, place, and person. Examination of cranial nerves turned out to be normal. Examination of the cerebellum revealed impaired finger nose test and dysdiadochokinesia, and heel-to-leg maneuver was also impaired. Romberg's test also turned out to be positive. A clinical diagnosis of ataxia was made. He was admitted and evaluated to discern the etiology. The patient underwent a routine laboratory checkup for complete blood count (CBC), liver function test (LFT), and renal function test (RFT). All investigations turned out to be normal. Magnetic resonance imaging (MRI) was done to rule out other lesions and were normal (Figure 1). TDM consultation was sought, and as per the advice of the toxicologist, the blood sample was analyzed for phenytoin levels using ultra high-performance liquid chromatography (UHPLC). It was slightly above the therapeutic range (30.5 mcg/ml). Normal phenytoin levels in blood range from 10 to 25 mcg/ml. Then, phenytoin was tapered to 100 mg BD and stopped completely in 3 weeks, after which there was a gradual improvement in ataxia. The patient was then switched over to valproate 200 mg twice daily. After three weeks, the patient started walking normally and had no further seizures after discontinuing phenytoin. The Naranjo scale was applied, and a score of 7 was obtained, suggesting probable ADR [7]. The Naranjo scoring of this particular case is given in Supplement 1.

## 3. Discussion

Phenytoin is a commonly used drug that is essential in epilepsy therapeutics. Phenytoin blocks sodium channels in the inactivated state, reducing neuronal excitability [4]. Despite the prescription of a usual adult dose, the patient developed manifestations similar to toxic doses. This emphasizes the importance of TDM and genetic testing in treating patients with epilepsy. Phenytoin is poorly water soluble and can irritate the vessels if infused. The metabolism occurs mainly through CYP2C9, which accounts for 80% of the process, and the remaining 20% is carried out by CYP2C19 [4]. The critical point here is the role of CYP2C19, which overtakes the function of CYP2C9 in the case of mutation that renders it nonfunctional [8]. The prevalence of CYP2C9 mutations and associated single nucleotide polymorphisms (SNPs) are found to be more common in the Indian and South Asian populations [5]. CYP2C9 polymorphisms make these people more vulnerable to phenytoin toxicity, even at normal therapeutic doses. Genetic polymorphism of CYP2C9 may reduce the metabolism of PHT by 25–50% in patients with variants ^*∗*^2 and ^*∗*^3 compared to those with wild-type variant ^*∗*^1 [6]. Therefore, utmost care has to be taken while administering phenytoin to patients from these population groups. Since testing for these single nucleotide polymorphisms (SNPs) may not be feasible in most low-resource settings, physicians and TDM consultants must emphasize careful monitoring and dose adjustment per the TDM values rather than relying on clinical response. Whenever possible, physicians must recommend genetic testing for CYP2C9 polymorphisms.

## 4. Conclusion

In this case, the supratherapeutic levels, despite standard dosing, were most likely attributed to CYP2C9 polymorphism, which is prevalent in Indians and other South Asians. The CYP2C9^*∗*^3 polymorphism is present in a significant proportion of the Indian people. This warrants genetic testing and employing TDM for meticulous monitoring of patients prescribed with phenytoin. Phenytoin is a common antiseizure medication. Physicians and neurologists must remember the underlying genetic components to produce optimal treatment benefits and reduce the incidence of such adverse effects.

## Data Availability

All data available are depicted in the case report and no additional data are available.

## Supplementary Materials

Supplementary Materials Supplement 1: Naranjo causality assessment and interpretation.
