# Supplementary material for: Ataxia and Seizures despite Phenytoin: A Case Report Highlighting the Importance of TDM and Genetic Influences
Source: Case Rep Neurol Med. 2024 Mar 20;2024:2888895. doi: 10.1155/2024/2888895 (PMC10977159; doi:10.1155/2024/2888895)
Supplement: Supplementary Materials — Supplement 1: Naranjo causality assessment and interpretation. [file 2888895.f1.docx]

| 1. Are there previous conclusive reports on this reaction? 1 |
| --- |
| 1. Did the adverse event appear after the suspected drug was administered? 2 |
| 1. Did the adverse reaction improve when the drug was discontinued or a   specific antagonist was administered? 1 |
| 1. Did the adverse event reappear when the drug was re‐administered? 0 |
| 1. Are there alternative causes (other than the drug) that could on their own   have caused the reaction? 0 |
| 1. Did the reaction reappear when a placebo was given? 0 |
| 1. Was the drug detected in blood (or other fluids) in concentrations known   to be toxic? 1 |
| 1. Was the reaction more severe when the dose was increased or less severe   when the dose was decreased? 1 |
| 1. Did the patient have a similar reaction to the same or similar drugs in any   previous exposure? 0 |
| 1. Was the adverse event confirmed by any objective evidence? 1 |
| Total score 7 |

**Supplement 1:**

**Naranjo scoring:**

| **Score** | **Interpretation of Scores** |
| --- | --- |
| **Total Score** **≥9** | **Definite**. The reaction (1) followed a reasonable temporal sequence after a drug or in which a toxic drug level had been established in body fluids or tissues, (2) followed a recognized response to the suspected drug, and (3) was confirmed by improvement on withdrawing the drug and reappeared on reexposure. |
| **Total Score** **5 to 8** | **Probable**. The reaction (1) followed a reasonable temporal sequence after a drug, (2) followed a recognized response to the suspected drug, (3) was confirmed by withdrawal but not by exposure to the drug, and (4) could not be reasonably explained by the known characteristics of the patient’s clinical state. |
| **Total Score** **1 to 4** | **Possible**. The reaction (1) followed a temporal sequence after a drug, (2) possibly followed a recognized pattern to the suspected drug, and (3) could be explained by characteristics of the patient’s disease. |
| **Total Score** **≤0** | **Doubtful**. The reaction was likely related to factors other than a drug. |
